# Enrichments of gene replacement events by *Agrobacterium*-mediated recombinase-mediated cassette exchange

**DOI:** 10.1007/s11032-015-0215-7

**Published:** 2015-02-15

**Authors:** Hiroyasu Ebinuma, Katsuhiko Nakahama, Kazuya Nanto

**Affiliations:** 1 Faculty of Textile Science and Technology, Shinshu University, 3-15-1, Tokida, Ueda, Nagano, 386-8567 Japan; 2 Agri-Biotechnology Research Laboratory, Nippon Paper Industries Co. Ltd., 5-21-1, Oji, Kita-ku, Tokyo, 114-0002 Japan

**Keywords:** RMCE, Gene replacement, Negative selection

## Abstract

We report recombinase-mediated cassette exchange (RMCE), which can permit integration of transgenes into pre-defined chromosomal loci with no co-expressed marker gene by using *Agrobacterium*-mediated transformation. Transgenic tobacco plants which have a single copy of negative marker genes (*codA*) at target loci in heterozygous and homozygous conditions were used for gene exchange by the RMCE method. By negative selection, we were able to obtain five heterozygous and four homozygous transgenic plants in which the genes were exchanged from 64 leaf segments of heterozygous and homozygous target plants, respectively. Except for one transgenic plant with an extra copy, the other eight plants had only a single copy of marker-free transgenes, and no footprint of random integrated copies was detected in half of the eight plants. The RMCE re-transformation frequencies were calculated as 6.25 % per explant and were approximately the same as the average percentage of intact single-copy transformation events for standard tobacco *Agrobacterium*-mediated transformation.

## Introduction

In current transformation methods, variable numbers of transgenes together with co-expressed marker genes are randomly inserted into the plant genome. Transgene instability and variation in expression levels are frequently caused by complex integration structure. A large amount of DNA and RNA analysis is required to identify transgenic plants with a single copy of transgenes with a stable expression level for analysis of gene functions and biotechnology risk assessments. Recent advances in recombinase-mediated cassette exchange (RMCE) have enabled enhanced tag-and-exchange strategies for single-copy high-throughput targeted integration (Turan et al. 2011, 2013). We have developed *Agrobacterium*-mediated transformation methods which enable us to introduce a marker-free transgene into pre-defined chromosomal loci by RMCE.

RMCE is a tool box for replacing a genomic target cassette with a compatible donor cassette. The target and donor cassettes are each flanked by two oppositely oriented recognition sites and recombinase catalyzes double-crossover between the two recognition sites for replacement. *Agrobacterium* introduces a small amount of linear single-stranded T-DNA, which is not a suitable substrate for recombinase, into the plant nucleus (Tinland et al. 1994). Before T-DNA integration, a transferred single-stranded T-DNA is converted into a double-stranded DNA (Singer et al. 2012). *Agrobacterium*-mediated methods are efficient for targeted transformation by RMCE (Nanto et al. 2005; Louwerse et al. 2007). A large difference in expression levels of exchanged genes is detected between four different chromosomal target sites (Nanto et al. 2009; Ebinuma et al. 2012). The concept of removing a marker gene from targeted transgenic plants is demonstrated by repeated transformation (Srivastava and Ow 2004; Nanto and Ebinuma 2008). Through multiple rounds of RMCE using biolistic-mediated transformation, groups of six transgenes are stacked onto the same target sites (Li et al. 2009, 2010).

Recently, designed sequence-specific nucleases (ZENs, TALENs, CRISPR/Cas) have been demonstrated to enable not only site-specific mutagenesis and integration but also gene replacement (Tzfira et al. 2012; Belhaj et al. 2013; Endo and Toki 2014). Gene targeting is generated by induction of double-strand breaks at specific genome loci and DNA repairs via non-homologous end joining (NHEJ) or homologous recombination (HR). Gene replacement events are reproduced via HR, and co-delivery of the gene of interest as a template for HR with nucleases to specific genome loci remains a problem in plants. We reported that *Agrobacterium* could efficiently co-deliver the gene of interest to target genome loci with recombinases and reproduce gene replacement events (Nanto et al. 2005). In this report, we demonstrate the enrichment protocols of gene replacement events by *Agrobacterium*-mediated RMCE and discuss the common problems of HR- and RMCE-mediated gene replacements.

## Results and discussion

### Molecular strategy

**Fig. 1:**
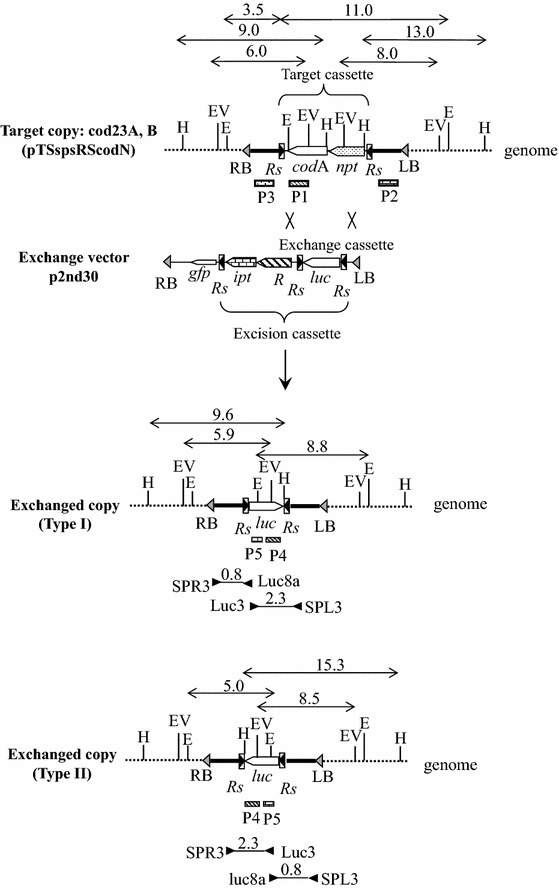
Molecular strategy for producing marker-free targeted transgenic plants by RMCE. When the exchange vector p2nd30 is introduced into target plants (cod23A, B), recombinase catalyzes double-crossover between the two *Rs* to replace the target cassette with the exchange cassette. As a result, the transgenic plants (Type I, Type II) that have an exchange cassette at the target locus are produced. The diagram shows the integrated T-DNA region containing a target cassette of target plants (cod23A, B), the T-DNA region of exchange vector p2nd30, the integrated T-DNA region containing an exchange cassette of transgenic plants (Type I, Type II), the position of probes (P1, P2, P3, P4, P5) for Southern analysis, the size (kb) of DNA fragments detected by the probes, restriction enzyme sites (E: *Eco*RI; EV: *Eco*RV; H: *Hind*III) and the DNA regions amplified with PCR primers (SPR3-Luc8a, Luc3-SPL3, SPR3-Luc3, Luc8a-SPL3). *codA* cytosine deaminase gene, *npt* neomycin phosphotransferase gene, *gfp* green-fluorescent protein gene, *ipt* isopentenyl transferase gene, *R* recombinase gene, *luc* firefly luciferase gene, *Rs* recognition site, *RB and LB* right and left border sequences of a T-DNA

We designed the site-directed integration (SDI) vector system for producing marker-free targeted transgenic plants. We used the site-specific recombination system (R/*Rs*) consisting of the recombinase (R) and its recognition sites (*Rs*), which is derived from the plasmid pSR1 of *Zygosaccharomyces rouxi* (Araki et al. 1992). The SDI vector system consists of a target vector to introduce a target cassette and an exchange vector to re-introduce an exchange cassette for gene replacement (Fig. 1). The target vector pTSspsRScodN has a target cassette containing target genes (*npt* and *codA*), which are flanked by two oppositely oriented *Rs*. The *codA* gene codes for cytosine deaminase, which converts 5-fluorocytosine (5-FC) to 5-fluorouracil (5-FU). Since 5-FU is toxic to plant cells, a transgenic plant with a *codA* gene is blighted on medium containing 5-FC (Schlaman and Hooykaas 1997). The exchange vector p2nd30 has an exchange cassette containing a desired gene (*luc*) flanked by two oppositely oriented *Rs*. Moreover, two directly oriented *Rs* flank the *ipt*, *R*, and *luc* genes (excision cassette), and the *gfp* gene is located outside the excision cassette. The *ipt* gene is derived from the Ti-plasmid of *Agrobacterium tumefaciens* and codes for isopentenyl transferase, which catalyzes cytokinin synthesis (Akiyoshi et al. 1984).

### Target line

**Fig. 2:**
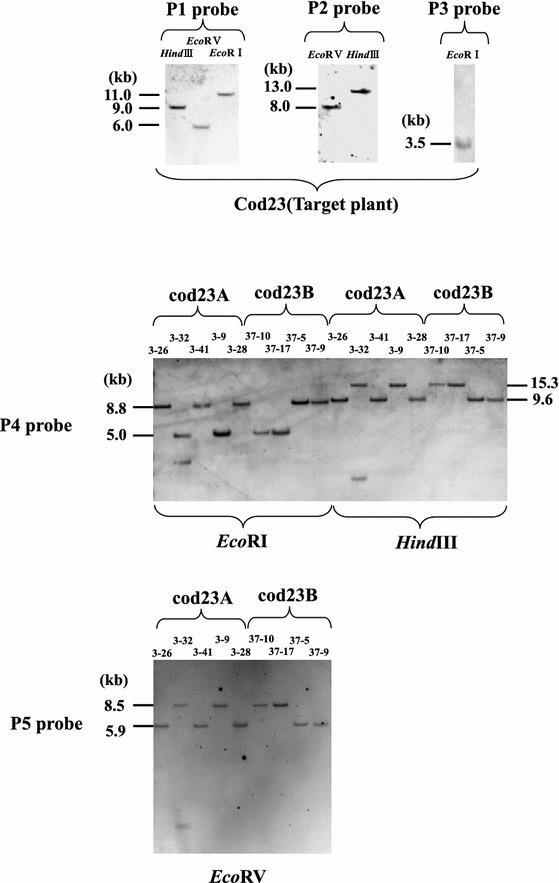
Southern analysis of T0 target plant (cod23) and transgenic plants in which the genes were exchanged from two target lines (cod23A, B). Genomic DNA was digested with three restriction enzymes (E: *Eco*RI; EV: *Eco*RV; H: *Hind*III) and hybridized with five probes (P1, P2, P3, P4, P5), respectively

We introduced the target vector pTSspsRScodN into tobacco plants and the T0 transgenic line (cod23) was self-crossed to produce T1 transgenic lines (cod23A, B) with a single copy of a target cassette in heterozygous and homozygous conditions. To confirm the copy number of target genes of cod23A and B lines, we isolated large-scale DNA from T0 transgenic plants (cod23) and digested it with three restriction enzymes for Southern analysis. The predicted 9.0-, 6.0- or 11.0-kb fragment was hybridized with the P1 probe (codA) by *Hind*III, *Eco*RV or *Eco*RI digestion, respectively. The predicted 8.0- or 13.0-kb fragment was hybridized with the P2 probe by *Eco*RV or *Hind*III digestion, respectively, and the predicted 3.5-kb fragment with the P3 probe by *Eco*RI digestion (Figs. 1, 2). These results indicate only the presence of a single copy of target genes in cod23 lines.

### Re-transformation

We infected 64 leaf segments from each of two target lines (cod23A, B) with *A. tumefaciens* containing the exchange vector p2nd30. First, we placed the infected leaf segments on the medium containing only plant hormones to induce callus formation. Two weeks after infection, we transferred these explants to the medium containing plant hormones and 5-FC for negative selection. After 1 month of cultivation, we separated 60 5-FC-resistant calluses from leaf segments and subcultured them on the same medium. After 1 month of cultivation, we separated less than two shoots from each callus and transferred them to hormone-free medium for rooting. Overall, about 6 months after infection, we obtained 40 normally rooted plants of independent callus lines from the cod23A line and 23 plants from the cod23B line.

### DNA analysis

To detect precise integration of the exchange cassette into the target locus, we isolated DNA from these normally rooted plants and subjected it to PCR analysis. When the target cassette was replaced by the exchange cassette, both the predicted 0.8- and 2.3-kb fragments were amplified with the primer pairs SPR3-Luc8a and Luc3-SPL3 (Type I), or the predicted 2.3- and 0.8-kb fragments were amplified with the primer pairs SPR3-Luc3 and Luc8a-SPL3 (Type II) (Fig. 1). In 40 normally rooted plants from the cod23A line, the expected exchange fragments were amplified in five plants, of which three were Type I and two were Type II. In 23 normally rooted plants from the cod23B line, the expected exchange fragments were amplified in four plants, of which two were Type I and two were Type II. Furthermore, we investigated the presence of the *npt*, *ipt* or *R* genes in these nine plants by PCR analysis. We detected the *luc* gene in all of the plants, but not the *npt*, *ipt* or *R* genes. We identified five plants from the cod23A line and four plants from the cod23B line as transgenic plants in which the genes were exchanged (Table 1).

**Table 1:** Transformation efficiency of target lines (cod23A, B) with the exchange vector p2nd30

Target lines	cod23A	cod23B
Explants^a^	64	64
Calluses^b^	60	60
Rooted shoots^c^	40	23
Transgenic plants^d^	5 (4)^e^	4 (4)^e^
Exchange frequency^f^ (%)	6.25	6.25

^a^Number of leaf segments infected with *Agrobacterium*

^b^Number of calluses cultured on the medium containing 5-FC

^c^Number of normally rooted shoots

^d^Number of transgenic plants which had an exchanged copy

^e^Number of transgenic plants which did not have any extra copies

^f^Percentage of transgenic plants which had only an exchanged copy

To determine the structure of the exchanged copy at the target locus, we isolated large-scale DNA from nine transgenic plants and digested it with three restriction enzymes for Southern analysis. In Type I transgenic plants, the predicted 8.8- or 9.6-kb fragment was hybridized with the P4 probe (*luc*) by *Eco*RI or *Hind*III digestion, respectively, and the predicted 5.9-kb fragment with the P5 probe (*luc*) by *Eco*RV digestion. In Type II transgenic plants, the predicted 5.0- or 15.3-kb fragment was hybridized with the P4 probe (*luc*) by *Eco*RI or *Hind*III digestion, respectively, and the predicted 8.5-kb fragment with the P5 probe (*luc*) by *Eco*RV digestion (Fig. 1). In all of the nine transgenic plants, the predicted exchange fragments were detected and in only one plant (3-32) was an extra fragment detected (Fig. 2). These results indicate that eight plants from the codA and B lines have only a single copy of the precisely exchanged cassette containing a desired gene (*luc*).

Furthermore, we investigated by PCR analysis the presence of the *gfp* gene in eight transgenic plants in which the genes were exchanged to confirm the footprints of randomly integrated copies on the plant genome. As the *gfp* gene was located outside the excision cassette flanked by two directly oriented *Rs* (Fig. 1), after the removal of randomly integrated copies by recombination, the *gfp* gene remained on the chromosome. We detected the *gfp* gene in one transgenic plant (3-26) from the cod23A line and three (37-10, 37-5, 37-9) from the cod23B line (data not shown). These results indicate that recombinase efficiently catalyzes the removal of randomly integrated copies and also the direct replacement of an exchange cassette with a target cassette at target loci, not through random integration.

### Progeny test

We backcrossed the wild tobacco plant SR1 with eight transgenic plants in which the genes were exchanged to investigate the segregation of an exchanged cassette containing a desired gene (*luc*). We obtained normal seeds from all transgenic plants and subjected T1 seeds to progeny tests by PCR analysis with the primer pairs Luc3–Luc8a. The progeny of four transgenic plants from the cod23A line were PCR-positive for the *luc* gene in the predicted segregation ratio (1:1). All of the progeny of four transgenic plants from the cod23B line were PCR-positive. These results indicate that the transgenic plants from the cod23A or B lines have a single exchanged copy in heterozygous or homozygous condition, respectively, and the exchanged copy is stably transmitted to the progeny.

### Expression of the luciferase (*luc*) gene

We subjected the progeny of eight transgenic plants in which the genes were exchanged from the cod23A and B lines to a quantitative luciferase assay. We analyzed five T1 plants from each transgenic plant and calculated the average luciferase activity. We detected high levels of luciferase activity in each of the transgenic plants. We report the production of eight control transgenic plants which contain a single copy of the transgenes, the *hpt* and *luc* genes, between two oppositely oriented *Rs* sites, by standard transformation methods (Nanto et al. 2009). Compared to exchanged lines, these lines clearly showed a high variability of luciferase activity.

### *Agrobacterium*-mediated RMCE

As we used *Agrobacterium* to re-introduce desired genes into target lines, we expected that most of them would be randomly integrated into the plant genome of target lines and that the probability with which recombinase exchanged a target gene for a desired gene at pre-defined genomic loci would be far too low. In this report, therefore, we employed several strategies with the aim of addressing these issues. The desired genes, together with the *ipt* and recombinase genes, are flanked by two directly oriented recognition sites (Fig. 1). Recombinase removes randomly integrated copies from the genome and the *ipt* gene is used as a negative marker to select transgenic plants without such copies. Moreover, the *codA* gene is located at the target loci and used as a negative marker to select targeted transgenic plants without it. We combine these strategies to enrich targeted events and to make screening procedures more simple and effective.

We located a *gfp* gene outside two directly oriented recognition sites, which flanked the desired genes, together with the *ipt* and recombinase genes (Fig. 1) in order to detect a footprint of their random integration into the genome. By PCR analysis, we detected no *gfp* gene in two of 13 targeted transgenic plants (Nanto et al. 2005), in one of two plants (Nanto and Ebinuma 2008), in five of 20 plants (Nanto et al. 2009) and in four of eight plants (this report). Such high percentages of targeted ones with no footprint indicate that a desired gene is directly exchanged with a target gene at pre-defined chromosomal loci by RMCE, not through random integration. It has been reported that a transferred single-stranded T-DNA is converted into double-stranded DNA before T-DNA integration (Singer et al. 2012). A double-stranded T-DNA intermediate is a good substrate for RMCE events by the SDI vector system.

### Promotion of RMCE events

We expected that two factors would play an important role in the efficiency of targeting events. One is the promotion effect by the *ipt* gene (Akiyoshi et al. 1984). The desired and recombinase genes are introduced together with the *ipt* gene into target lines. The *ipt* gene would increase the frequency of gene targeting events during transformation since cytokinin induces callus formation and proliferates selectively transgenic cells where the desired and recombinase genes are introduced. The other is the high paring ability of recognition sites. We use the site-specific recombination system (R/*Rs*) derived from the plasmid pSR1 of *Zygosaccharomyces rouxii* (Araki et al. 1992). Their plasmids contain a pair of the 959-bp inverted repeats, within a 58-bp region on which recombinase catalyzes site-specific recombination. This region contains a 7-bp spacer sequence flanked by two 12-bp inverted repeat sequences. One of these two repeats is flanked by four additional direct repeats with 4-bp spacer sequences. By PCR, we amplified a 400-bp recognition site containing all these repeats from the 959-bp inverted repeats. In the bacteriophage P1 Cre/*lox* system, a recognition site in which two 13-bp inverted repeat sequences flank an 8-bp spacer sequence is widely used (Wang et al. 2011). As the paring ability of recognition sites depends on size and structure, a 400-bp sequence may promote the alignment of a desired gene with a target gene and increase the exchange frequency.

### HR- and RMCE-mediated gene replacements

Unlike NHEJ-mediated site-specific mutagenesis and deletion, HR-mediated gene replacement needs co-delivery of the gene of interest as a template for HR with nucleases to specific genome sites. Gene replacement events using ZFNs (Wright et al. 2005; Cai et al. 2009; Townsend et al. 2009; Shukla et al. 2009; de Pater et al. 2009, 2013; Qi et al. 2013) and TALENs (Zhang et al. 2013) were detected but transgenic plants were obtained in only a few studies (Shukla et al. 2009; de Pater et al. 2009, 2013) because the technical demand is high. Most studies co-delivered the template DNA and nuclease expression constructs to cell suspensions or protoplasts by direct DNA transformation methods. They make it possible to introduce a large quantity of DNA into large populations of plant cells and identify the gene replacement events without large-scale selection, whereas conventional *Agrobacterium*-mediated transformation methods, which are commonly used for a wide variety of crops, introduce a small quantity of single-strand T-DNA into small populations of plant cells and integrate it randomly into the plant genome. In *Arabidopsis*, transgenic plants with gene replacement events via the floral dip transformation method were obtained with frequencies of 1.0 × 10^−3^ (de Pater et al. 2009) and 3.1 × 10^−3^ (de Pater et al. 2013) per transformation event, and the authors employed gene-specific selection regimes in which HR-mediated modifications of pre-inserted or endogenous genes confer a selectable phenotype. The positive–negative selection regime can be used for any target gene but relies on the generation of very large number of transformation events. Thus, development of selection regimes and enrichment protocols is needed for screening of the cells including the HR-mediated gene replacement events from the non-transgenic cells, and the transgenic cells including the NHEJ-mediated mutagenesis events or the randomly integrated events. In this study, we employ the “hit-and-run” cassette strategy of the MAT vector system (Ebinuma et al. 2005) which enables enrichments of gene replacement events by removal of randomly integrated copies. The “hit-and-run” cassette has the *ipt* and recombinase genes which are flanked by two directly oriented recognition sites. The *ipt* gene promotes the increase in transgenic cells and recombinase removes randomly integrated copies from the genome. The timing of expression of recombinase can be tightly harmonized with the co-delivery of the gene of interest. As a result, RMCE-mediated gene replacement events are concentrated. The “hit-and-run” cassette strategy is simple and effective and applicable to HR-mediated gene replacement.

## Materials and methods

### Plasmid constructs

Plasmids were constructed using standard recombinant techniques (Maniatis et al. 1982). The binary vector plasmid pTSsps contained an *Sse*8387I site between *Sma*I and *Sac*I at a specific sequence. The pCAmp plasmid contained an *amp* gene flanked by two oppositely oriented *Rs* sequences (Nanto et al. 2005). The *Hind*III fragment of the *nos* promoter-*npt*-*nos* terminator and the *Hind*III-*Eco*RI fragment of the CaMV35S promoter-*codA*-*nos* terminator were ligated into the *Hind*III site and the *Hind*III-*Eco*RI sites of pCAmp to produce pCAmpcodN. pCAmpcodN was reconstructed to produce pCcodN by removing the *amp* gene. The *Sse*8387I fragment of pCcodN was ligated into the *Sse*8387I site of pTSsps to produce the target vector pTSspsRScodN (Fig. 1).

The *Hind*III-*Sma*I fragment of the *Rs* sequence was amplified by PCR and ligated into the *Hind*III-*Sma*I site of pTL7 to produce pTLRS(sma). The *Hind*III fragment of the CaMV35S promoter-*gfp*-*nos* terminator, the *Kpn*I fragment of the *Rbc*-3B promoter-*ipt*, and the *Eco*RI fragment of the CaMV35S promoter-*R*-*nos* terminator were ligated into the *Hind*III, *Kpn*I, and *Eco*RI sites of pTLRS(sma) to produce pTLRSRubipt35R. The fragment of the *nos* promoter-*luc*-*nos* terminator was ligated into the *Kpn*I sites of pCAmp to produce pCAmpluc. pCAmpluc was reconstructed to produce pCluc by removing the *amp* gene. The *Sse*8387I fragment of pCluc was ligated into the Sse8387I site of pTLRSRubipt35R to produce the exchange vector p2nd30 (Fig. 1).

### Plant transformation

The binary vector plasmid pTSspsRScodN was introduced into *A. tumefaciens* strain LBA4404 by electroporation (Nagel et al. 1990). Leaves of in vitro clones of *Nicotiana tabacum* cv. SR1 were cut into small pieces (8 mm square), inoculated with *A. tumefaciens* in dilute overnight culture medium (OD_630_ = 0.25), and blotted dry with sterile filter paper. The explants were placed on MS agar medium containing 40 mg/L acetosyringone for 3 days and transferred to MS agar medium containing 1 mg/L benzyladenine, 0.1 mg/L naphthaleneacetic acid, 500 mg/L carbenicillin, and 200 mg/L kanamycin. After 1 month of cultivation, calluses were separated from the leaf segments and subcultured on the same medium. After 1 month of cultivation, regenerated shoots were separated from the calluses and transferred to hormone-free MS agar medium containing 200 mg/L cefotaxim. Transgenic plants that rooted normally were propagated in vitro for DNA analysis. The single-copy transgenic line (cod23) was selected on the basis of genetic and Southern blot analysis (Fig. 2). The T0 transgenic line (cod23) was self-crossed to produce T1 transgenic lines (cod23A, B) with a single copy of a target cassette in heterozygous and homozygous conditions. The segregation of kanamycin resistance in their progeny (T2) was investigated to identify their copy number of target cassettes.

Leaf segments of in vitro clones of transgenic plant lines (cod23A, B) were inoculated with *A. tumefaciens* containing p2nd30. The explants were placed on MS agar medium containing 40 mg/L acetosyringone for 3 days and cultured on MS agar medium containing 1 mg/L benzyladenine, 0.1 mg/L naphthaleneacetic acid, and 500 mg/L carbenicillin for 2 weeks. The explants were then transferred to MS agar medium containing 1 mg/L benzyladenine, 0.1 mg/L naphthaleneacetic acid, 500 mg/L carbenicillin, and 200 mg/L 5-fluorocytosine. After 1 month of cultivation, calluses were separated from the leaf segments and subcultured on the same medium. After 1 month of cultivation, regenerated shoots were separated from the calluses and transferred to hormone-free MS agar medium containing 200 mg/L cefotaxim. Transgenic plants that rooted normally were propagated in vitro for DNA analysis.

### PCR and Southern analysis

Genomic DNA was isolated from leaves of in vitro transgenic plants using a FastDNA Kit, following the supplier’s instructions (Bio 101, Qbiogene, Carlsbad, CA, USA). PCR was carried out under standard conditions with 30 cycles of 30 s of denaturation at 94 °C, 30 s of annealing at 60 °C, and 2 min of extension at 72 °C. Reaction products were resolved by electrophoresis on 1.2 % (w/v) agarose gel. The sequences of the PCR primers (Fig. 1) were as follows:

SPR3: 5′-TTACTTTGCGTTTGTGTACT-3′;
SPL3: 5′-TGCTTTTGCGTCTGCCATTG-3′;
Luc3: 5′-CGTTCGGTTGGCAGAAGCTATGAAA-3′;
Luc8a: 5′-TTTCATAGCTTCTGCCAACCGAACG-3′.

Genomic DNA was isolated from leaves of in vitro transgenic plants by a modified CTAB method. Ten micrograms of DNA were digested with appropriate restriction enzymes, separated on 0.8 % (w/v) agarose gel and blotted onto a nylon membrane (Hybond-N, Amersham). After UV crosslinking, the membrane was hybridized with a DIG-labeled probe. The probe DNA fragment was labeled by PCR using DIG-dUTP, following the supplier’s instructions (Boehringer Mannheim). PCR primers for labeling were as follows:

P1 probe: 5′-TAACGCTTTACAAACAATTA-3′
  and 5′-CGCCCCGTTATAGGAGTGCA-3′;
P2 probe: 5′-ACATAAGATGATACGCAAGC-3′
  and 5′-CATTGCGGACGTTTTTAATGTACTG-3′;
P3 probe: 5′-GTTTACCCGCCAATATACCTGTCA-3′
  and 5′-TCATGTGTTTGCGTTTCATT-3′;
P4 probe: 5′-CATCACGGTTTTGGAATGTTTACTA-3′
  and 5′-CGGAGGATTACAATAGCTAAGAATT-3′;
P5 probe: 5′-CGTTCGGTTGGCAGAAGCTATGAAA-3′
  and 5′-AGGTGCGCCCCCAGAAGCAATTCG-3′

Hybridization, washing, and detection were performed using DIG Easy Hyb (hybridization solution) and a DIG Wash and Block Buffer Set (Roche, Penzberg, Germany) following the supplier’s instructions.

### Luciferase assay

Luciferase activity was assayed in five expanded leaf extracts after undergoing the various treatments described using the Pica Gene luciferase assay kit (Toyo-Inc., Tokyo, Japan). The protein concentration of the extract was determined using the Bio-Rad Protein Assay kit (Bio-Rad, Hercules, CA, USA). The light intensity from 1 μg of the protein extract was measured using a Luminescenser-JNR (ATTO, Tokyo, Japan) for 10 s, and is represented as the relative luciferase activity (RLA/μg).

## Abbreviations

RMCE: Recombinase-mediated cassette exchange
SDI: Site-directed integration
5-FC: 5-Fluorocytosine
5-FU: 5-Fluorouracil
CTAB: Cetyltrimethylammonium bromide
